# Perioperative neurocognitive functioning in elderly patients undergoing awake craniotomy for high grade glioma

**DOI:** 10.1093/noajnl/vdag009

**Published:** 2026-02-17

**Authors:** Eric A Goethe, Kyle R Noll, Subhiksha Srinivasan, Dima Suki, Sujit S Prabhu, Jeffrey S Weinberg, Ian E Mccutcheon, Chibawanye I Ene, Frederick F Lang, Shiao-Pei S Weathers, Catherine Sullaway, Mary F Mcaleer, Jeffrey S Wefel, Sherise D Ferguson

**Affiliations:** Department of Neurosurgery, The University of Texas MD Anderson Cancer Center, Houston, TX, USA; Department of Neuro-Oncology, The University of Texas MD Anderson Cancer Center, Houston, TX, USA; Department of Neurosurgery, The University of Texas MD Anderson Cancer Center, Houston, TX, USA; Department of Neurosurgery, The University of Texas MD Anderson Cancer Center, Houston, TX, USA; Department of Neurosurgery, The University of Texas MD Anderson Cancer Center, Houston, TX, USA; Department of Neurosurgery, The University of Texas MD Anderson Cancer Center, Houston, TX, USA; Department of Neurosurgery, The University of Texas MD Anderson Cancer Center, Houston, TX, USA; Department of Neurosurgery, The University of Texas MD Anderson Cancer Center, Houston, TX, USA; Department of Neurosurgery, The University of Texas MD Anderson Cancer Center, Houston, TX, USA; Department of Neuro-Oncology, The University of Texas MD Anderson Cancer Center, Houston, TX, USA; Department of Neuro-Oncology, The University of Texas MD Anderson Cancer Center, Houston, TX, USA; Department of Radiation Oncology, The University of Texas MD Anderson Cancer Center, Houston, TX, USA; Department of Neuro-Oncology, The University of Texas MD Anderson Cancer Center, Houston, TX, USA; Department of Radiation Oncology, The University of Texas MD Anderson Cancer Center, Houston, TX, USA; Department of Neurosurgery, The University of Texas MD Anderson Cancer Center, Houston, TX, USA

**Keywords:** awake craniotomy, cognition, elderly, glioma, ­neuropsychology, outcome

## Abstract

**Introduction:**

Older patients may be at particular risk of decline in neurocognitive function (NCF) following brain tumor resection, particularly when tumors are near-eloquent regions.

**Methods:**

We identified 95 patients of advanced age (≥60 years) with newly diagnosed, left hemisphere, high-grade eloquent glioma who underwent a first-time awake craniotomy for tumor resection. All patients had comprehensive neuropsychological evaluations preoperatively and a subset of patients (*N* = 45) completed postoperative assessment.

**Results:**

Median age at surgery was 66 years (range, 60-81) and tumors were most commonly located in the temporal (56%) and frontal (27%) lobes. Preoperatively, most patients exhibited NCF impairment on at least 1 neuropsychological test, most frequently in verbal learning (66%) and memory (71%). Localization in temporal regions conveyed greater impairment to memory, and patients with frontal tumors exhibited poorer executive functioning. Following awake resection, neurocognitive decline was most frequent in verbal learning (41%) and memory (38%). Postoperative change in neurocognitive performance was not associated with more advanced age, comorbidities, frailty, or tumor volume or extent of resection.

**Conclusions:**

Advanced age, frailty, and medical comorbidities were not significantly associated with poorer NCF outcome. Postoperative decline was greatest when patients present with high levels of baseline functioning and tumors involve the mesial temporal lobe. Importantly, rates of baseline impairment and postoperative decline in this older age sample were similar to other cohorts, including younger patients and lower grade tumors. Findings support the relative safety of awake craniotomy for resection of eloquent glioma in advanced age individuals.

Key Points

- Elderly patients with dominant hemisphere gliomas present with a variety of neurocognitive deficits but most commonly in verbal learning and memory.
- Most elderly patients who undergo resection of dominant hemisphere glioma experience neurocognitive decline following surgery though rates of decline are similar to that of younger cohorts, and more advanced age within the elderly does not appear to convey further risk of postoperative worsening.

Importance of the Study

The role of aggressive treatment of glioma in elderly patients was historically downplayed, often because it was considered futile or because patients were not considered able to withstand the procedure. While it has become clear that oncologic benefit can be obtained from standard therapy in elderly patients, little is known about the neurocognitive burden of glioma and its management in elderly patients, including whether advanced age and frailty in this vulnerable group is associated with poorer outcome following resection. As early postoperative neurocognitive outcomes are strongly linked to survival, this prognostic information is critical for patients, caregivers, and physicians alike. Our study shows that most elderly patients have impaired neurocognitive function at presentation and decline following surgery at similar rates to younger cohorts. We also identify preoperative neurocognitive characteristics and tumoral features strongly predictive of worse outcomes following awake eloquent glioma resection which may aid prognostication.

Treatment of high-grade gliomas typically involves maximal safe resection followed by chemoradiation and adjuvant chemotherapy.^1^ Glioma patients frequently exhibit a wide range of deficits in neurocognitive functioning (NCF) due to mass effect, direct parenchymal invasion, and/or seizures that can disrupt broad brain networks underlying NCF.^2^ Tumor resection also conveys risk to NCF, especially when lesions are located near-eloquent brain.^3^^,^^4^ While awake resection informed by preoperative and intraoperative brain mapping can help mitigate risk of surgically acquired decline in some critical functions, especially aspects of language, a substantial proportion of patients exhibit postoperative worsening despite use of these modern imaging and surgical techniques.^5^ While patients typically recover some functioning over time, a considerable proportion do not return to their baseline levels.^6^^,^^7^ Importantly, NCF impairment is associated with reduced quality of life,^8^ functional independence,^9^ and even survival^10^ in patients with glioma. These associations underscore the need to preserve NCF, especially given the very limited survival duration of this aggressive disease. Identification of risk factors associated with surgically acquired NCF decline may improve preoperative counseling of patients and caregivers, as well as point to potential groups of patients in which development of further brain mapping, surgical planning, and other mitigation approaches may be most needed.

Historically, elderly patients have often been excluded from consideration of aggressive surgery.^3^ This has been particularly true for elderly patients harboring tumors suspected to be high-grade, located in eloquent cortex, and surgery involving awake craniotomy, due to assumptions regarding reduced tolerability and potentially limited benefit from aggressive resection.^11^ Accordingly, less is known about the NCF profiles of these patients and effects of surgery. Despite a paucity of NCF outcome data, accumulating evidence over the past few decades indicates that elderly patients can tolerate surgical management of glioma, including awake surgery.^12^^,^^13^ This study delineates the preoperative NCF baseline of elderly patients with high-grade glioma in eloquent regions and characterizes the effects of awake resection upon NCF outcomes. Contributors to impairment and postoperative decline are also explored, including patient demographic, clinical, and tumoral characteristics. We also examine relationships between age, frailty, and cognition in this elderly sample to better understand whether risk of impairment and decline increase with more advanced age and number of comorbidities in this group of patients often presumed be particularly high-risk. Better understanding of relationships between these clinical and patient characteristics and NCF outcomes may improve patient counseling and risk stratification, including identification of those in which more comprehensive brain mapping may be considered to help mitigate potential decline.

## Methods

### Participants

Adult patients with high-grade glioma were retrospectively identified who met the following criteria: (1) at least 60 years of age at time of resection; (2) left hemisphere tumors within eloquent or near-eloquent regions per Sawaya et al;^14^ (3) baseline neuropsychological evaluation prior to tumor resection; (4) underwent awake craniotomy; and (5) histopathological confirmation of high-grade glioma (grade 3/III or 4/IV) per WHO criteria at the time of presentation. Patients with history of a prior open resection (biopsy allowed), radiotherapy, or any systemic therapy prior to completion of baseline neuropsychological testing were excluded. Ninety-five patients met criteria and were included in the study. Of these, 45 also had postoperative neuropsychological evaluations. The University of Texas MD Anderson Cancer Center Institutional Review Board approved this study.

### Clinical Characteristics

Clinical variables were extracted from medical records, including KPS scores, speech deficit or memory complaint per clinical exam at preoperative neurosurgery visit, preexisting comorbidities, length of postoperative hospital stay, discharge disposition, and medication use. Intraoperative mapping technique and complications were also recorded. The five-item modified frailty index (mFI-5)^15^ was calculated for each patient as the total of the following comorbidities: congestive heart failure, chronic obstructive pulmonary disease, dependent functional status (KPS ≤ 60), diabetes, and hypertension. In glioma, higher scores are associated with poorer outcomes, including postoperative complications, discharge other than home, 30-day readmission, and survival.^16^

### Volumetrics and Tumor Characteristics

Tumor volumes were determined with MedVision (Evergreen Technologies, Inc., Castine, Maine, United States) or Vitrea software (Canon Medical Informatics, Inc., Minnetonka, Minnesota, United States) as previously described.^17^ Trained departmental data management research staff aided delineation of tumor boundaries. Volumetrics and extent of resection (EOR) were derived from pre- and 24- to 48-hour postoperative MRI. For the majority of tumors which were primarily enhancing, T1 post gadolinium-contrast hyperintensity was used, while hyperintensity on T2/FLAIR sequences was used for the 7 predominantly non-enhancing lesions. For tumors extending beyond a single lobe, location of the majority of the mass (>50%) was coded. Primarily insular and basal ganglia locations were also noted. Extension into the insula, mesial temporal lobe (ie, hippocampus, uncus, and/or amygdala), and subcortical structures (eg, caudate, basal ganglia, internal/external capsule, thalamus) were also recorded. Extension characteristics were coded in a binary fashion (yes/no) based upon either enhancing or non-enhancing disease infiltration by the first author and adjudicated, if necessary, by the senior author. All MRIs were also reviewed by a board-certified neuro-radiologist.

### Neurocognitive Testing

Neurocognitive testing was conducted as part of standard of care perioperative evaluations by trained psychometrists under the supervision of board-certified clinical neuropsychologists. Presurgical and postsurgical neuropsychological evaluations occurred within a month prior to surgery (*M* = 4.3 days, SD = 4.5) and about 2 months following baseline evaluation (*M* = 53.8 days, SD = 42.0). Postoperative testing occurred prior to adjuvant treatment, except for 5 patients who were evaluated following completion of chemoradiation. When available, alternate forms were utilized at postsurgical evaluation to minimize practice effects. Clinic practices and updates to tests evolved over time resulting in minor differences in tests used within domains of interest. Table 1 lists the neuropsychological tests by domain routinely included in the assessment and number of patients completing each preoperatively and postoperatively. The number administered a given test slightly differed by instrument, as evaluations utilized a flexible battery for clinical purposes. Test scores were standardized using demographically adjusted normative data^3^^,^^18–26^ and converted into *z*-scores (*M* = 0, SD = 1). Scores of −1.5 or lower were considered impaired.^3^ As previously described,^3^ differences between presurgical and postsurgical *z*-scores that ranged from −1.00 to −1.99 were considered mildly declined, changes of −2.00 to −2.99 moderately declined, and −3.00 or lower severely declined.

**Table 1.** Cognitive tests grouped by principal domain and number of completers.

Domain	Test	Abbreviation	Preop *N*	Postop *N*
Attention	WAIS-R/III/IV Digit Span	Digit span	87	41
Memory	HVLT-R Total Recall/RAVLT Immediate	Learning	87	38
	HVLT-R Delayed Recall/RAVLT Delay	Delayed recall	77	33
	HVLT-R Recognition Discrimination/	Recognition	76	32
	RAVLT Recognition Hits			
Processing speed	WAIS-R/III/IV Digit Symbol/Coding	Coding	85	38
	Trail Making Test Part A	TMTA	89	41
Executive function	Trail Making Test Part B	TMTB	81	37
	WAIS-R/III/IV Similarities	Similarities	80	34
	MAE Controlled Oral Word Association	COWA	93	38
Language	MAE Token Test	Token	72	30
	MAE Visual Naming or Boston Naming Test	Naming	87	37
Visuospatial	WAIS-R/III/IV Block Design	Block design	78	35

Abbreviations: WAIS-R, Wechsler Adult Intelligence Scale-Revised; WAIS-III, Wechsler Adult Intelligence Scale-Third Edition; WAIS-IV, Wechsler Adult Intelligence Scale-Fourth Edition; HVLT-R, Hopkins Verbal Learning Test-Revised; Rey Auditory Verbal Learning Test; MAE, Multilingual Aphasia Examination.

### Statistical Analysis

Associations between baseline NCF *z*-scores and preoperative to postoperative *z*-score changes with clinical characteristics were determined with Spearman rank-order (ρ) and point-biserial (rpb) correlations, and independent samples *t*-tests, as appropriate. Effect sizes of group differences were described with Cohen’s d.^27^ Multivariate stepwise binary logistic regression investigated predictors of preoperative NCF impairment (present vs absent) and preoperative to postoperative NCF change (decline vs not). Significant characteristics from univariate analyses were included as predictors. Penalized likelihood (Firth’s method) was utilized to stabilize model estimates when quasi-complete or complete separation was present. All analyses were performed with SPSS 30.0 (IBM Corp.) with extension in R implemented for Firth’s method. Given the exploratory nature of the investigation, two-sided tests were used with an unadjusted significance level of *P* ≤ .05.

## Results

### Demographic and Clinical Characteristics

Patient characteristics for the total sample and subset that completed postoperative neuropsychological evaluation are presented in Table 2. For the overall sample, median age was 66 years, and most tumors were glioblastoma (GBM, 86%) and located within the temporal (56%) or frontal (27%) lobes. Comorbidities were common (80%), though frailty on the mFI-5 was infrequent, with 38% of patients having a total score of 1 and only 9% of the sample with scores of at least 2. The awake portion of surgery was terminated before completion of resection in 9 patients (discomfort or poor compliance with mapping in 8; seizure in 1). Intraoperative direct electrical stimulation language mapping was performed in 93% of cases. Intraoperative speech decline was reported in 33%. Median extent of resection (EOR) was 100% with 77% achieving gross total resection. Patient characteristics did not significantly differ between those with and without postoperative neuropsychological evaluations.

**Table 2.** Demographic and clinical characteristics for the total and postoperative outcome samples.

	Overall	Outcome
	*N* = 95	*N* = 45
Age in years		
Mdn (IQR)	66.0 (8.0)	66.5 (8.5)
Range	60-78	60-78
% Male	55	52
% White	86	86
% Right hand dominant	90	93
Education, years		
Mean (SD)	15.2 (2.9)	15.9 (2.6)
Range	7-20	7-20
Comorbidities		
% yes	80	77
Total, Mean (SD)	2.3 (1.9)	2.1 (2.1)
Modified Frailty Index		
Mdn (IQR)	0.0 (1.0)	0.5 (1.0)
Range	0-3	0-3
Preop speech deficit, % yes	68	59
Preop memory complaint, % yes	33	44
Seizure, % yes	28	33
Medications		
Any, % yes	98	95
Total, Mean (SD)	5.1 (2.7)	5.1 (3.0)
Steroid, % yes	65	58
Antiepileptic, % yes	61	65
Histology, %		
Glioblastoma	86	81
Astrocytoma	9	16
Oligodendroglioma	4	3
Other	1	0
WHO tumor grade^a^		
% Grade 3/III	14	18
% Grade 4/IV	86	82
Hemisphere, %		
Left	100	100
Primary location, %		
Temporal	56	66
Frontal	27	27
Parietal	12	7
Insular/basal ganglia	4	0
Occipital	1	0
Infiltration, %		
Insula	30	25
Mesial temporal	15	18
Subcortical	7	7
Tumor volume^a^,cm^3^		
Mean (SD)	29.8 (22.0)	28.2 (23.3)
Range	0.4-93.7	0.4-92.8
Preoperative FLAIR volume^b^, mm^3^		
Mean (SD)	61.5 (43.4)	63.1 (53.4)
Range	4.2-216.7	4.2-216.7
Awake craniotomy, %	100	100
Cortical stimulation, %		
Language	93	93
Motor	36	18
Extent of resection^a^, %		
Mdn (IQR)	100 (64.32)	100 (59.71)
>90, %	82	84
Therapy at follow-up, %		
Chemotherapy and/or radiation	5	9
KPS		
Preoperative ≥ 80, %	92	96
Postoperative ≥ 80, %	86	88
Postop Change, Mdn (IQR)	0.0 (10.0)	0.0 (0.00)
Length of stay		
Days, Mean (SD)	5.6 (6.4)	5.3 (5.3)
Disposition, %		
Home	88	91
Rehabilitation	9	7
Skilled nursing	2	3
Other	1	0

a *N* = 94; T1 postcontrast hyperintense volume for enhancing tumors, T2/FLAIR hyperintense volume for nonenhancing lesions.

b *N* = 94.

### Preoperative Neurocognitive Functioning

At the battery-wide level, preoperative impairment was observed on at least 1 test in 89% of patients, with 63% impaired on 3 or more tests, and 43% on at least 5 tests. Mean performances and rates of NCF impairment on individual tests are presented in Table 3. Impairment rates ranged from 4% to 66%, most frequent in memory (learning, 66%; delayed recall, 70%), and executive functioning (TMTB, 52%; COWA, 63%). These tests were also most severely impaired on average, with mean *z*-scores ranging from −1.75 (COWA) to −2.64 (Delayed Recall). Older age was not significantly associated with worse preoperative performances. Higher education was associated with better abstract reasoning (similarities: ρ = 0.29, *P* < .001).

**Table 3.** Preoperative and postoperative neurocognitive performances.

	Preoperative			Preoperative to postoperative change		
Domain and test	*N*	*z*-score Mean (SD)	% Impaired	*N*	z-score Mean (SD)	% declined
*Attention*						
Digit span	87	−0.78 (1.03)	17	41	−0.33 (2.00)	30
*Learning and memory*						
Learning	87	−2.18 (1.50)	66	38	−2.41 (2.89)	42
Delayed recall	77	−2.64 (1.94)	70	33	−2.88 (4.09)	39
Recognition	76	−1.50 (1.95)	40	32	−2.71 (2.73)	36
*Processing speed*						
Coding	85	−0.31 (0.92)	15	38	−0.67 (2.33)	9
TMTA	89	−1.35 (3.33)	34	41	−1.00 (2.33)	42
*Executive function*						
TMTB	81	−3.58 (5.32)	52	37	−1.10 (4.95)	53
Similarities	80	−0.52 (1.08)	24	34	−0.67 (1.83)	31
COWA	93	−1.75 (1.31)	63	38	−2.10 (2.26)	40
*Language*						
Token	72	−0.56 (1.39)	32	30	−0.44 (2.15)	32
Naming	87	−1.01 (1.53)	40	37	−1.30 (2.50)	34
*Visuospatial function*						
Block design	78	−0.11 (0.94)	4	35	−0.17 (1.42)	13

See Table 1 for abbreviations.

Comparison of baseline NCF by lobe was restricted to frontal versus temporal given the small number of tumors in other regions. Frontal lesions exhibited worse executive functioning [COWA: d = −0.61; *P* = .013) and temporal tumors showed poorer memory (recognition: d = −0.52; *P* = .036]. Involvement of the mesial temporal lobe was also related to poorer memory [delayed recall: d = −0.75; *P* = .020; recognition: d = −0.87; *P* = .007]. Greater tumor volume was associated with worse performance on most tests, but most strongly in language (Naming: ρ = −0.50; *P*<.001), executive functioning (COWA: ρ = −0.56; *P* < .001; TMTB: ρ = −0.52; *P* < ,001), and memory (Learning: ρ = −0.49; *P* < .001; Delayed Recall: ρ =−0.39; *P* < .001). Higher preoperative FLAIR volume was also associated with worse performance on most tests, especially for memory (Learning: ρ = −0.61; *P* < .001), language (Naming: ρ = −0.56; *P* < .001), and aspects of executive functioning (COWA: ρ = −.64, *P* <  .001; Similarities: ρ = −.58, *P* < .001). As expected, presence of midline shift and mass effect were highly colinear (ρ = .93; *P* < .001) but associated with worse performance on numerous tests, most strongly in language (Token) and executive functioning (COWA), but to a lesser magnitude than volumetric measures (ρ = −.26 to −.38; all *P* < .05). Correlations between NCF performance and tumor imaging characteristics are presented in Supplementary Table S1.

Preoperative steroid treatment was associated with worse NCF across measures of attention (Digit Span: d = −0.69; *P* = .003), aspects of executive functioning (Similarities: d = −0.55; *P* = .020), language (naming: d = −0.70; *P* = .003; Token: d = −0.66; *P* = .006), visuoconstruction (Block Design: d = −0.62; *P* = .011), memory (Learning: d = −0.98; *P* < .001; Delayed recall: d = −0.79; *P* = .001), processing speed (TMTA: d = −0.42; *P* = .019), and aspects of executive functioning (TMTB: d = −0.55; *P* = .019). Worse NCF was not associated with higher tumor grade, presence or number of comorbidities, frailty score, seizure status, antiepileptic use, or total number of medications. Poorer NCF was also not associated with the rarely occurring subcortical or insular infiltration.

Higher preoperative KPS was associated with better performances on most NCF tests, most strongly in executive functioning (ρ = .34 to .41), memory (ρ =.24 to .43), and processing speed (ρ =.35; all *P* < .05). Preoperative self-reported memory complaint was significantly associated with poorer object naming aspects of language (Naming: d = −0.53; *P* = .021), and preoperative speech deficit on neurologic exam was significantly associated with reduced attention (Digit Span: d = −0.73; *P* = .002), aspects of executive functioning (COWA: d = −0.48; *P* = .034), and visuoconstruction (Block Design: d = −0.51; *P* = .037). Baseline performance was not associated with intraoperative speech decline, postoperative length of stay, or disposition (home vs other).

### Prediction of Preoperative Neurocognitive Impairment

Predictive models of preoperative impairment on frequently impaired tests (>30% of the sample) were explored. Predictors drawn from univariate analyses above included tumor and FLAIR volume, midline shift or mass effect (yes/no), steroid use (yes/no), temporal (yes/no) and frontal (yes/no) localization, and mesial temporal lobe involvement (yes/no). Despite expected relationships, steroid treatment was not associated with tumor volume or presence of midline shift and/or mass effect, supporting their inclusion as independent predictors. Results of significant predictive models are summarized in Table 4. Presence of baseline impairment on learning and memory tests was best predicted by steroid use, greater lesion (FLAIR) volume, presence of mass effect or midline shift, and temporal lobe localization and involvement of mesial structures, with final models accounting for 36% to 51% of variance. Impairment in mental flexibility (TMTB) and verbal fluency (COWA) aspects of executive functioning were predicted by larger FLAIR volume (33% to 55% of variance accounted), while impaired basic processing speed (TMTA) was modestly predicted by FLAIR volume and location outside the temporal lobe (*R*^2^ = 0.22). Language impairment in naming was best predicted by tumor volume (contrast-enhancing) and temporal lobe localization (48% variance explained).

**Table 4.** Predictors of preoperative neurocognitive impairment on selected tests.

Neuropsychological test	Predictor(s)	Coefficient^a^	Standard Error	OR	*p*-value	*R* ^2^
Learning						
Model 1	Steroid	2.26	0.57	9.58	<.001	0.28
Model 2	Steroid	2.12	0.84	8.35	<.001	0.51
	FLAIR volume	0.05	0.02	1.05		
Delayed Recall						
Model 1	Steroid	1.49	0.58	4.44	.009	0.14
Model 2	Steroid	1.52	0.61	4.58	.002	0.25
	Mass effect/shift^b^	2.18	1.48	8.84		
Model 3	Steroid	1.61	0.64	5.00	<.001	0.36
	Mass effect/shift^b^	2.62	1.49	9.58		
	MTL involvement^b^	2.02	0.95	7.53		
Recognition						
Model 1	FLAIR volume	0.03	0.01	1.03	<.001	0.21
Model 2	FLAIR volume	0.04	0.01	1.04	<.001	0.39
	Temporal involvement	2.17	0.70	8.73		
Model 3	FLAIR volume	0.04	0.01	1.04	<.001	0.46
	Temporal involvement	2.00	0.75	7.35		
	MTL involvement	2.00	0.95	7.06		
TMTA						
Model 1	Temporal involvement	−1.37	0.51	0.26	.005	0.13
Model 2	Temporal involvement	−1.41	0.53	0.24	.001	0.21
	FLAIR volume	0.02	0.01	1.02		
TMTB						
Model 1	FLAIR volume	0.13	0.03	1.14	<.001	0.55
COWA						
Model 1	FLAIR volume	0.04	0.01	1.04	<.001	0.33
Naming						
Model 1	Tumor volume	0.07	0.02	1.08	<.001	0.41
Model 2	Tumor volume	0.08	0.02	1.09	<.001	0.48
	Temporal involvement	1.49	0.64	4.46		

Note. MTL, mesial temporal lobe; see Table 1 for test abbreviations.

a Coefficients represent B for maximum and β for penalized (Firth) partial likelihood estimates.

b Firth’s penalized partial likelihood approach used for estimates.

### Postoperative Neurocognitive Change

**Figure 1.**
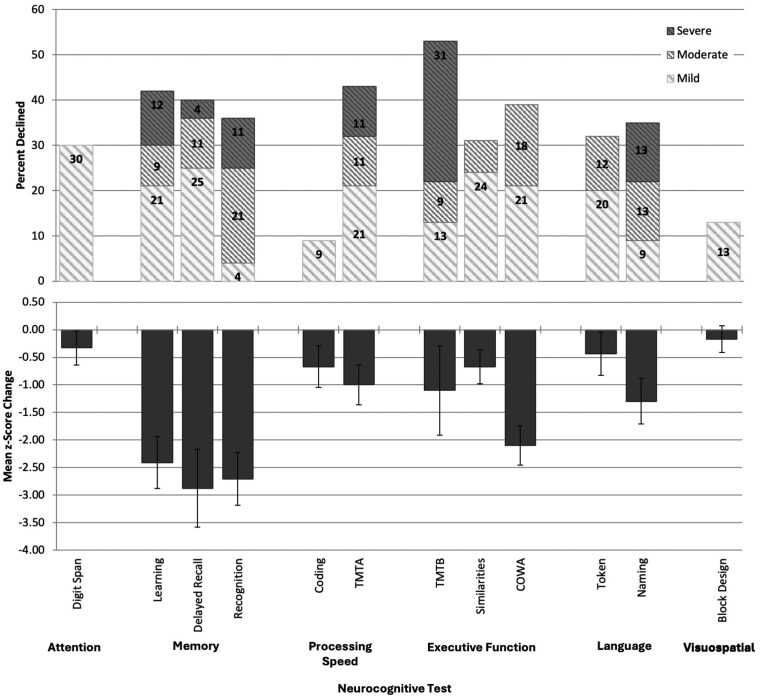
Postoperative neurocognitive change across tests. Top, rates of decline. Severe decline, *z*-score change < −3.00; Moderate decline, *z*-score change −2.99 to −2.00; Mild decline, *z*-score change −1.99 to −1.00. Bottom, preoperative to postoperative change scores. See Table 1 for sample size descriptions. Error bars represent standard error of the mean.

At the battery-wide level, clinically meaningful preoperative to postoperative decline was observed on at least 1 test in 72% of patients, with 51% declining on 3 or more tests, and 27% on at least 5 tests. As described in Table 3 and depicted in the top panel of Figure 1, rates of individual decline ranged from 9% to 53%, most frequently in memory (36% to 42%) and executive functioning (31% to 53%). Greatest decline was noted in these same domains, with severe declines frequent in memory (Learning, 12%; Recognition, 11%) and executive functioning (TMTB, 31%), but also in processing speed (TMTA, 11%) and language (Naming, 13%). Mean change in NCF test *z*-scores is presented in Table 3 and illustrated in lower panel of Figure 1. All tests showed negative mean *z*-score changes at the group level, most prominently in memory (*M* = −2.41 to −2.88), executive functioning (TMTB M = −1.10 COWA M = −2.10), and language (Naming: *M* = −1.30). Older age was only associated with poorer outcome on a single measure of processing speed (Coding: ρ=−0.44; *P* = .011). Education, sex, ethnicity, and test-retest interval were not associated with outcomes. Comorbidities, frailty, and medication use, type, and number were not associated with outcomes.

NCF change did not differ by frontal versus temporal location. However, involvement of the mesial temporal region was related to poorer outcome in memory (Delayed Recall: *d* = −0.60; *P* = .039) and executive functioning (TMTB: *d* = −0.86; *P* = .044; Similarities: *d* = −1.19; *P* = .015). Nearly all with follow-up data did not have subcortical involvement and few had mass effect and/or midline shift. Greater preoperative tumor volume was associated with better outcome in language and verbal fluency/executive functioning (Naming: ρ = .52; *P* = .003; COWA: *ρ* = 0.56; *P* < .001). Greater preoperative FLAIR volume was also associated better outcome in COWA (ρ = .50; *P* = .001). Neither preoperative NCF nor intraoperative speech decline was related to EOR, and EOR was not associated with postoperative NCF outcomes.

Of patients with postoperative NCF testing, 91% were discharged home, though shorter length of hospital stay was significantly associated with better outcomes in aspects of executive functioning (Similarities: ρ= −0.52; *P* = .004), language comprehension (Token: ρ = −0.41; *P* = .044), and memory (Learning: ρ = −0.39; *P* = .024). While higher preoperative KPS was associated with worsening in a component of memory (Recognition: ρ = −0.38; *P* = .044), change in KPS was not associated with NCF outcomes. In contrast with the rates of decline noted on NCF testing, only 5% exhibited worsening of KPS greater than 10.

### Prediction of Postoperative Neurocognitive Decline

The utility of baseline performance and tumor characteristics for predicting postoperative decline on tests with frequent decline (>30%) was explored. Tumoral predictors included preoperative tumor volume and presence of extension in the mesial temporal lobe given univariate associations with outcomes. Results of significant predictive models are summarized in Table 5. Preoperative Delayed Recall memory accounted for 39% of variance in this outcome, which improved to 52% when including infiltration into the mesial temporal lobe. Recognition memory, verbal fluency aspects of executive functioning (COWA), and language comprehension (Token) outcomes were also predicted by preoperative baselines, which accounted for 26%, 28%, and 39% of variance, respectively. Outcome in Naming aspects of expressive language was predicted by baseline, which accounted for 29% of variance and increased to 50% when including mesial temporal involvement. For all significant models, higher preoperative NCF was associated with increased risk of postoperative decline.

**Table 5.** Predictors of neurocognitive decline on selected tests.

Neurocognitive outcome	Predictor(s)	Unstandardized B	Standard error B	OR	*P*-value	*R* ^2^
Delayed recall						
Model 1	Baseline	0.65	0.25	1.92	.002	0.39
Model 2	Baseline	0.99	0.38	2.68	.001	0.52
	Mesial temporal	−2.85	1.63	0.06		
Recognition						
Model	Baseline	0.70	0.37	2.00	.015	0.26
COWA						
Model 1	Baseline	0.83	0.34	2.30	.003	0.28
Naming						
Model 1	Baseline	0.81	0.35	2.24	.006	0.29
Model 2	Baseline	1.17	0.47	3.22	.001	0.50
	Mesial temporal	−3.04	1.36	0.05		
Token						
Model 1	Baseline	1.19	0.59	3.29	.004	0.39

*Note*. See Table 1 for abbreviations and sample sizes.

Follow-up analyses explored whether patients with better preoperative performances remained at a higher level of postoperative NCF than those with lower baselines, despite showing more risk of clinically significant decline. This was examined with one-way analysis of variance, with postoperative *z*-score as the dependent variable and preoperative *z*-score tertiles groups comprising independent factors (ie, 3 levels of baseline NCF). Mean postoperative *z*-scores did not significantly differ according to level of baseline NCF (all *P* > .05). Although not significant, an average difference of about 1 SD in postoperative NCF was observed according to baseline tertile. That is, those with baseline scores in the middle and lowest tertiles had postoperative scores about 1 and 2 SDs below those with highest baseline.

## Discussion

Elderly patients comprise a large proportion of those with malignant glioma.^28^ Older age is associated with greater adverse effects of all surgeries upon NCF, with over half of elderly patients showing some postoperative decrement irrespective of the operative site. Glioma surgery adds complexity, as tumor location, mass effect, seizures, and medication-related side effects can contribute to NCF impairment, both at baseline and in the postoperative setting.^2^ Eloquent region tumors are associated with particularly severe NCF impairment at presentation and risk of decline following resection, even when surgery is performed under awake conditions.^5^^,^^8^ Importantly, preoperative and postoperative NCF deficits are associated with poorer quality of life,^8^ greater disability,^9^ and reduced survival,^10^ with baseline functioning appearing prognostic of outcome.^29^ The short survival period for most patients with malignant glioma also highlights the importance of predicting postoperative NCF outcomes and mitigating impairment.^2^ While elderly patients are often assumed to be more vulnerable to the impact of eloquent glioma and awake surgery upon NCF than younger individuals, it is recognized that age alone does not suffice for surgical decision-making and should be contextualized by disease characteristics, resection goals, patient health status, and level of functioning at presentation.^11^^,^^30^

### Preoperative Neurocognitive Impairment is Common in Elderly Glioma Patients

Estimates indicate over 90% of patients with glioma may exhibit NCF impairment at baseline, which represents a common presenting symptom in elderly and younger patients alike.^31–33^ A high rate of preoperative dysfunction was observed in our elderly eloquent glioma cohort, with some differences in NCF profile according to lesion location and infiltration. Localization in the frontal lobe was related with greater impairment in verbal fluency aspects of executive functioning, while those with lesions in the temporal lobe showed more memory dysfunction, particularly when tumors infiltrated mesial temporal structures. This is consistent with known brain-behavior relationships and confirms prior work regarding tumor localization and patterns of NCF.^3^^,^^34–36^ Additionally, larger lesion volume, presence of mass effect or midline shift, and steroid use were associated with worse NCF across multiple domains. Combinations of these factors accounted for about 50% of variance in aspects of memory, language (naming), and executive functioning, suggesting larger more symptomatic lesions are more prone to disrupt broad networks underlying diverse functions.^4^^,^^37^

Given that a majority of patients had GBM diagnoses, which represents a histopathology with typical onset in early to mid-60s, the pattern and prevalence of NCF impairment described may be considered generally representative newly diagnosed GBM patients. However, the rather wide variability in age range in our elderly sample allowed specific interrogation of whether advancing age within this suspected to be vulnerable group conveys incremental increase in risk to NCF. Notably, within this already elderly sample, older age was not associated with worse preoperative NCF, and broadly, rates and patterns of impairment appeared similar to other cohorts of dominant hemisphere malignant glioma patients, including our prior work involving younger samples.^3^^,^^5^ About 80% of patients had medical comorbidities, 30% had seizures, and an average of 5 medications were prescribed per patient, though these clinical characteristics were not related to NCF. Similarly, frailty scores were not related to preoperative NCF; however, the cohort was relatively robust per the mFI-5 and baseline KPS. In contrast with our prior work investigating tumor grade and NCF,^38^ we did not observe greater impairment in those with higher-grade tumors. However, this likely reflects the fact that only high-grade tumors were included in the present study, of which 86% were grade 4/IV limiting ability to make comparisons with the grade 3/III group.

### Neurocognitive Decline following Glioma Resection

At follow-up of about 2 months after awake craniotomy and tumor resection, a majority of patients (∼70%) showed clinically significant decline on at least 1 test and half worsened on at least 3 measures. This finding is in stark contrast with KPS, which remained generally stable. This suggests limited sensitivity of the KPS to less overt functional deficits, including those important for independence in instrumental activities such as higher-order NCF.^3^^,^^9^ Despite broad NCF decline, severity and rates of worsening varied considerably by domain. Consistent with prior work in eloquent glioma resection,^5^ executive functioning and memory appeared most vulnerable to postoperative deterioration, though slowing in processing speed was also common. Notably, around a third of patients declined in naming aspects of expressive language, despite all undergoing awake craniotomy for monitoring of function, with surgery informed by results of intraoperative direct electrical stimulation language mapping in 93% of the sample.

Most experts contend that patients undergoing glioma resection tend to show a V-shaped trajectory in NCF, with an initial postoperative decline followed by improvement over 3-6 months.^29^^,^^39^ Test-retest interval was not significantly associated with NCF change in our sample, though it is acknowledged that some of the cohort may have exhibited improvement with more time for recovery. However, this possibility must be tempered by the additional risks to NCF associated with the adjuvant therapies required beginning in the weeks after surgery, in addition to tumor progression. This latter factor represents the primary consideration for selecting the somewhat brief postoperative follow-up interval, as the majority of the sample constituted patients with GBM, many of which would have progressed and exhibited associated NCF deterioration at longer-term follow-up. Nonetheless, even in the relatively early postoperative period, identification of risk factors and mitigation strategies for postoperative NCF decline remains an important goal in this population. In fact, our data indicate that those with less postoperative decline have shorter hospital stays, and perhaps as a consequence, a more expedient return to independence in daily life and improved quality life in the context of a disease with poor survival duration. Additionally, understanding and reducing risk of early postoperative NCF decline may offer a higher “set-point” for rehabilitation, potentially hastening recovery trajectories in those with acquired deficits.

Advanced age was only associated with postoperative change on a single measure of processing speed, suggesting a limited relationship between older age and NCF outcome within this already elderly cohort. Broadly, the rates and patterns of postoperative decline agree with prior work involving dominant hemisphere and eloquent glioma resection across broader age distributions and tumor types.^5^^,^^40^ Additionally, no significant associations were identified between NCF outcome and medical comorbidities, medications, or frailty. This contrasts with other studies suggesting that older age and frailty represent risk factors for worse NCF, performance status, and prognosis.^10^^,^^38^^,^^41^ This discrepancy may relate to the fact that most patients in our cohort exhibited relatively high functional status and low levels of frailty and our findings may best inform surgical risks for patients with similar presenting characteristics.

Baseline NCF was strongly associated with postoperative outcomes. Higher preoperative NCF appeared predictive of postoperative decline in memory, verbal fluency aspects of executive functioning, and naming and comprehension aspects of language. Regarding memory and naming outcomes, predictive models appeared strongest when including mesial temporal involvement alongside preoperative NCF, consistent with the roles of these structures in memory storage and semantic network functions.^39^^,^^42^ These results are consistent with Hendi et al., in which up to 75% of patients with intact baseline NCF declined following eloquent glioma resection compared with around 60% in those with preoperative deficits.^43^ However, Santini et al.^44^ reported a lower risk of postoperative decline associated with better preserved preoperative NCF. While both studied outcomes following awake craniotomy, Hendi et al. included only high-grade tumors, whereas mostly grade 2/II tumors comprised the cohort of Santini and colleagues. Potentially, the comparatively slower tumor progression in the lower-grade group may permit cortical reorganization of function away from peritumoral regions, allowing for safer resection regardless of baseline function.

Despite potential differences across tumor grades, the relationship between preoperative NCF and risk of decline may seem somewhat intuitive. Those with higher preoperative NCF may simply have “more to lose,” and despite large magnitude declines, could nonetheless remain with better postoperative function than their counterparts with poorer baseline. Exploring this possibility, we found a trend in which, at postoperative evaluation, those with higher baseline NCF did remain slightly above the level of those with lower baseline. However, differences were non-significant and small. Indeed, mean postoperative NCF fell well below the level of impairment in the high baseline group, indicating substantial and clinically significant impairment in these patients at follow-up. Together, these data support the utility of outcome prediction models involving baseline NCF ­status, as patients with higher baselines appear at greatest risk of postoperative NCF decline, and resulting declines constitute a level of NCF impairment in the postoperative period at a magnitude potentially impacting functional independence.

While confirmation is needed, results may inform clinical decision-making and counseling in patients similar to our cohort. Specifically, the contention that elderly patients necessarily represent poor candidates for awake craniotomies and advanced age entails worse NCF outcome is not supported by our findings. However, the sizeable proportion of patients exhibiting decline in language functioning despite use of intraoperative mapping stresses the continued need for improved strategies for mitigating risk of NCF decline, including but not limited to the language domain. More nuanced approaches to brain mapping (eg, multimodal/multi-paradigm) may be especially of consideration for patients presenting with higher baseline functioning, given potential to complete more complex paradigms during mapping and elevated risk of postoperative decline. Further, those presenting with mesial temporal tumors may benefit from preoperative counseling regarding possible elevated risk of postoperative changes in memory and language abilities, as well as close monitoring postoperatively given associations between impairment in these domains and reduced functional independence.^9^

Notably, preoperative and postoperative tumor volumetrics and EOR did not exhibit consistent or substantial associations with NCF outcomes. Limited associations between EOR and NCF in awake eloquent glioma resection have been previously reported, though the literature is inconsistent.^42–44^ Nonetheless, the lack of association in the present study may also reflect a restriction of range, given that median EOR was 100%. Accordingly, further work would help clarify relationships between EOR and NCF outcomes, especially within elderly cohorts.

### Limitations

It is acknowledged that referral bias may impact the generalizability of our findings, as cases were clinically referred for preoperative neuropsychological evaluation. Accordingly, very low functioning patients at baseline incapable of participating in evaluation were likely not referred, and consequently, excluded from study. Similarly, only a subgroup of patients completed postoperative follow-up neuropsychological testing, which may reflect practical considerations (eg, pursuing care elsewhere, declining testing) but also potentially an inability to participate in testing. This observation raises the possibility that the frequency and magnitude of NCF decline following awake craniotomy may be higher than observed in our study. We also note that follow-up occurred approximately 2 months following surgery and may not fully capture patients at completion of recovery.^45^ Larger prospective studies remain needed, including patients with greater differences in baseline health status and observations involving longer-term follow-up intervals with a uniform neuropsychological test battery utilized. Future work would also benefit from investigation of potential moderating factors, such as premorbid intellectual functioning and social determinants of health. It should also be noted that our sample was predominantly comprised of patients with frontal and temporal tumors. Finally, as described above, our results are broadly consistent with prior studies involving glioma of the dominant hemisphere; however, these investigations also involved predominantly frontal and temporal^3^^,^^5^ or insular^46^ tumor cohorts. While glioma are most commonly found in the frontal and temporal regions, we acknowledge that much less is known about associations between more posteriorly located tumors and NCF outcomes. As such, further work should strive to incorporate greater diversity of lesion locations and generalization of our findings must consider the context of our sample characteristics.

## Conclusions

Awake craniotomies can be performed in elderly patients with high-grade eloquent glioma with excellent EOR and postoperative outcomes largely comparable to more general glioma cohorts. Importantly, within the elderly population, advancing age does not appear to convey incremental risk to NCF at baseline or change following resection. However, despite well-preserved performance status after surgery, a majority of patients exhibit NCF decline persisting several weeks to months following resection. In addition to being among the issues reported as most distressing to patients with glioma,^31^ these NCF changes can hinder quality of life and independence during a limited survival period. With improved identification of those at greatest risk of NCF decline, it is hoped that patient counseling can be better informed, and mitigation approaches better targeted and further developed, which may include more comprehensive brain mapping (nTMS, ECoG, and fMRI) and targeted prehabilitation or rehabilitation approaches.

## Supplementary Material

vdag009_Supplementary_Data

## Data Availability

The data used in the present study will be made available upon reasonable request.

## Previous Presentation

This work was previously presented as a poster at the 2024 Annual Scientific Meeting of the Congress of Neurological Surgeons in Houston, Texas.
