# Supplementary material for: Perioperative neurocognitive functioning in elderly patients undergoing awake craniotomy for high grade glioma
Source: Neurooncol Adv. 2026 Feb 17;8(1):vdag009. doi: 10.1093/noajnl/vdag009 (PMC12978308; doi:10.1093/noajnl/vdag009)
Supplement: vdag009_Supplementary_Data [file vdag009_supplementary_data.docx]

**Supplementary Material**

Table 1. Associations between tumor imaging characteristics and preoperative neurocognitive functioning

| **Domain and Test** | **Tumor Imaging Characteristic** | | | | | | |
| --- | --- | --- | --- | --- | --- | --- | --- |
|  | **Volumetric (ρ)** | | **Extension (rpb)** | | | | |
|  | Tumor | FLAIR | Insula | MTL | Subcortical | | Mass Effect/Midline Shift |
| *Attention*  Digit Span | **-.47** | **-.46** | .07 | .20 | | **.22** | -.20 |
| *Learning and Memory*  Learning  Delayed Recall  Recognition | **-.56**  **-.40**  **-.33** | **-.61**  **-.47**  **-.34** | .02  .16  .21 | -.01  **-.26**  **-.27** | | .20  .15  .08 | **-.23**  -.12  -.16 |
| *Processing Speed*  Coding  TMTA | **-.26**  **-.39** | **-.36**  **-.36** | .08  .11 | -.01  .09 | | .07  .03 | **-.26**  -.10 |
| *Executive Function*  TMTB  Similarities  COWA | **-.59**  **-.45**  **-.59** | **-.36**  **-.58**  **-.64** | .09  .04  -.06 | .06  .13  .**26** | | .07  .18  **.30** | -.05  **-.30**  **-.35** |
| *Language*  Token  Naming | **-.47**  **-.54** | **-.49**  **-.56** | .10  .09 | **.26**  -.09 | | **.30**  .12 | **-.38**  **-.39** |
| *Visuospatial Function*  Block Design | -.21 | -.20 | .15 | .21 | | .22 | -.02 |

Note. MTL, mesial temporal lobe; Tumor Volume represents predominantly contrast enhancing tumor; see Table 1 for test abbreviations.

Bolded, significant p < .05.
